# *Fas Ligand (FasL)* in Association with *Tumor-Infiltrating Lymphocytes (TILs)* in Early Stage Cervical Cancer

**DOI:** 10.31557/APJCP.2020.21.3.831

**Published:** 2020-03

**Authors:** Tricia Dewi Anggraeni, Primariadewi Rustamadji, M Farid Aziz

**Affiliations:** 1 *Department of Obstetrics and Gynecology, *; 2 *Department of Anatomic Pathology , Faculty of Medicine, University of Indonesia, Cipto Mangunkusumo Hospital, Indonesia. *

**Keywords:** Cervical cancer, FasL, TIL, apoptosis

## Abstract

**Objective:**

To date, little is known about the roles of FasL and TILs in cervical cancer. This study aims to determine the correlation between *FasL* expression and *TILs* presence in cervical cancer.

**Methods:**

In this study, we analysed the *FasL* and *TIL* presence in 32 squamous cell carcinoma or adenocarcinoma that were obtained from early stage (≤ IIA2) cervical cancer patients using immunohistochemistry. The level of *FasL* and* TIL* was assessed qualitatively, and then quantified with the H-Score system.

**Results:**

Most of the patients were between 30 to 50 years old (59,4%), and had never taken pap smear examination before (96,9%). Based on the Pearson analysis of *FasL* and *TIL* presence, we found that *FasL* was inversely correlated with *CD45* or *TIL* number when the level of *FasL *is above 140 and the *CD45* is below 160. Based on Chi-Square test of *FasL* and *TIL *classification, there was a nine-fold odds ratio (OR) of lower *TILs* classification in high expression of FasL classification (OR 9, p=0.01).

**Conclusion:**

An inverse correlation between *FasL* expression and *TILs* level, that might indicate *FasL*-induced *TILs* apoptosis in tumor tissue, was observed. The strong inverse correlation between *FasL* and *TILs* presence showed some insight about the interactions between cancer cells and its surroundings inside of the cervical cancer tissue. This might also be further developed to tailor a prognostic marker that can predict the outcome of therapy in patients, not only in cervical cancer, but generally in all cancer.

## Introduction

According to GLOBOCAN 2008, cervical cancer is the third most common cancer in the world and the fourth most common cause of mortality in women (Ferlay et al., 2013). The incidence of cervical cancer in 2008 was 471,000 cases, with 288,000 mortalities. High morbidity and mortality of cervical cancer is more commonly encountered in developing countries compared to developed countries. In Indonesia, cervical cancer is the most common cancer as consistently reported in 1990-1999 and 2005-2008. Cervical cancer is still a common problem and burden for the government due to high morbidity, high mortality, and high cost of treatment (Suzanna, 2009).

Cancer is a state of disease where tissue grows uncontrollably, and one of its hallmarks is the resistance of programmed cell death or apoptosis (Hanahan and Weinberg, 2000, 2011). Normally, if an unrepairable error were found in a cell or a group of cells, the system will drive the cells through apoptosis that may be induced intrinsically, such as through mitochondrial oxidative stress induction, and extrinsically, which mainly initiated by the contact of Fas receptor protein with its ligand, Fas ligand (*FasL*) (Lowin et al., 1994; Winter et al., 1999). *FasL* is a type II membrane protein and tumor necrosis factor (TNF) that could activate T-lymphocytes or Natural Killer (NK) cells leading to the apoptosis of Fas expressing cell (Walczak and Krammer, 2000).

However, in the state of cancer, *FasL* induction is not effective since most cancer cells are resistant to apoptosis (Peter et al., 2015). Furthermore, upregulation of *FasL* in cancer cells may worsen the prognosis by causing ‘counterattack’ against natural killer (NK) cells and tumor infiltrating lymphocytes (*TILs*) which are responsible as antitumor effector cells (Chappell and Restifo, 1998; Igney et al., 2000; O’connell et al., 1996).

The ‘counterattack’ effect can also be observed through the decline of tumor-infiltrating lymphocytes (*TILs*) in different kind of tumor tissues with FasL overexpression, such as colon (O’Connell et al., 2000), esophageal (Bennett et al., 1998), breast (Ioachim et al., 2005), and oral cancer (Fang et al., 2013). *TILs* are suspected to be a prognostic factor for cancer metastases, progression into advanced stages, and relapse incidence (de Jong et al., 2009). However, the association between *FasL* overexpression and T-lymphocytes is still controversial. A study on cervical carcinoma did not find any significant association between *FasL* expression and* TILs* levels (Munakata et al., 2005). In Indonesia, a previous study confirmed a significant association between *FasL *expression and lymph nodes metastases in patients with early stage cervical cancer who underwent primary surgery (Irwanto, 2016).

To date, little is known about the roles of *FasL* and *TILs* in cervical cancer. This study aims to determine the correlation between *FasL* expression and *TILs* presence, due to the described controversy, in early stage cervical cancer tissues. In the future, this study will lead to identification of high-risk cervical cancer with lymph node involvement and to improve the treatment options for cervical cancer management.

## Materials and Methods

*Study design and Sampling*

This cross-sectional study was approved by the Ethical Committee at Faculty of Medicine Universitas Indonesia – Cipto Mangunkusumo Hospital (CMH), Jakarta. Forty three paraffin embedded squamous cell carcinoma or adenocarcinoma of cervical cancer samples were retrospectively and consecutively obtained from January 2007 to May 2011 in the Division of Gynecologic Oncology, Department of Obstetrics and Gynecology, and Department of Anatomical Pathology at CMH. Forty three samples were obtained from early stage (stage ≤ IIA2) cervical cancer patients based on the International Federation of Gynecology and Obstetrics (FIGO) classification, who underwent primary radical hysterectomy at the same period at CMH. Eleven tissue specimens were excluded due to lacking of tumor cells. Thirty-two samples were subjected to the examination of *FasL* and *TIL* levels by *IHC*. 

*Immunohistochemistry*

For the *IHC* staining, 4 µm sections were made and processed according to the protocol of previous study. *FasL *and *CD45+* cells (*TIL*) were detected using Thermo Fisher Scientific *CD178/Fas* Ligand Antibody (Thermo Fisher Scientific, QB1980882, USA) and Novocastra Lyophilized Mouse Monoclonal Antibody *CD45 *(Leica Biosystems, NCL-LLA, USA), respectively. Meanwhile, the primary antibodies were recognized by the secondary antibody Horseradish Peroxidase-labeled (HRP) septravidine/Trekavidine-HRP (Biocare Medical, STHRP700 L10, USA). Detection kit used in this study was Biocare Detection System (Biocare Medical, LLC, Concord CA, USA). IHC staining was performed according to the product manual (Irwanto, 2016).

Each slide was randomly observed on five visual fields; each visual field was divided onto nine smaller fields to be analyzed (see supplement 1). Analysis was performed independently using imageJ software. The level of FasL and TIL was assessed qualitatively and divided into four stages, a strong positive (given the value +3), moderate positive (+2), weak positive (+1), and negative (-). After qualitative assessment, quantitative calculations were performed using the formula H-Score (Białas et al., 2003; Ishibashi et al., 2003).

*Statistical Analysis*

All data were collected in a computerized database and statistically analyzed by SPSS 18.0 [SPSS Inc., Chicago, USA]. Bland-Altman analysis was performed to analyze the variance of the quantification between two observer. There was an agreed analysis guide between the two observers (pathologist as first observer and researcher as second observer) which was statistically proven by Bland-Altman analysis (see supplement 2). Correlation analysis was conducted using Pearson analysis to obtain cut-off-point of FasL (high and low expression) and TILs (high and low level). Data were analyzed by independent T-Test (Dahlan, 2011).

## Results

*Patients Characteristic and Demographic*

Biopsy samples were obtained from a total of 32 patients with cervical cancer. The demographic and disease characteristics are shown in Table 1 below. Most of the patients were between 30 to 50 years old (59.4%), had married between the age of 16-24 years (56.3%), were married once (90.6%), had experienced childbirth mostly before reaching 25 years old (87.5%), and had delivered 1-2 children (59.4%). Contraceptive use was not a common practice among the patients since there was still 43.8% that never had contraception. Most of the patients (96.9%) had never taken pap smear examination before, and only 1 patient (3.1%) ever had pap smear examination. 

*FasL Expression and TIL Levels in Cervical Cancer Biopsy*

**Figure 1:**
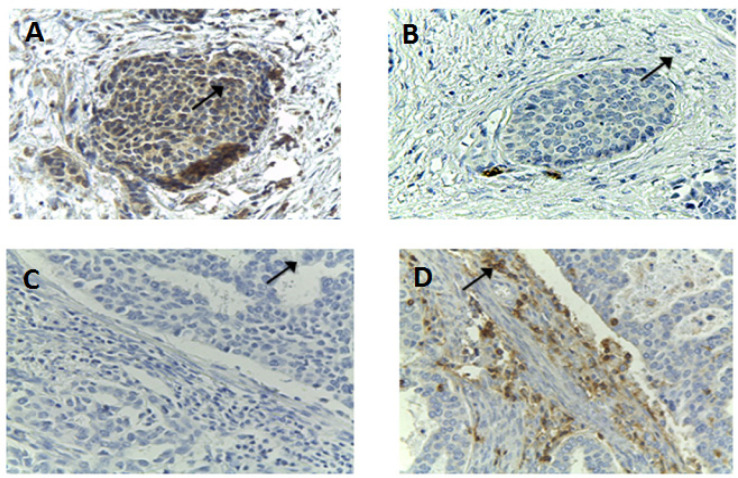
The Coloration of *FasL* and *TIL* Using *IHC* Staining. The sections were stained with antibody against fasL (A and C), and antibody against TIL (B and D). A and B were taken from the same section, so are C and D

In order to elucidate the correlation between *FasL* expression and *TIL* presence in cervical cancer tissue, the levels of *FasL* and *TIL* were investigated using IHC. The levels of *FasL *and *TILs* are depicted in Figure 1. On every adjusted section, FasL high expression (Figure 1A) led to the decreased coloration of *TILs*-immunohistochemical staining cells (Figure 1B), and, accordingly, low expression of *FasL* (Figure 1C) was accompanied with high presence of* TILs* (Figure 1D). 

**Figure 2:**
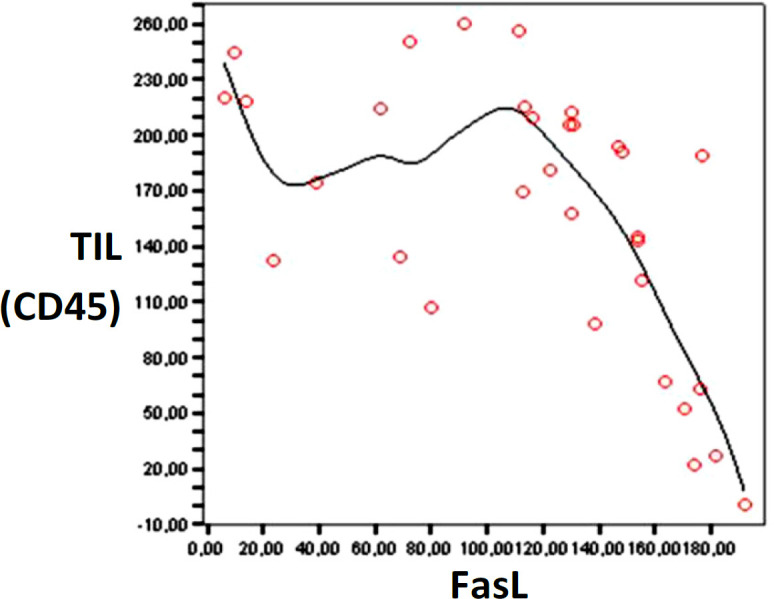
The Pearson Analysis between *FasL* and *TILs *Showed Inversed Correlation when the FasL is Above 140 and the *TIL* amount is below 160

Based on Pearson analysis, we found that *FasL* was inversely correlated with *CD45* or *TIL* number when the level of *FasL *is above 140 and the CD45 is below 160 (as shown in Figure 2). Based on that observation, we categorized the expression of *FasL* as ‘high’ if the H-score was more than 140, otherwise it was classified as low. Similarly, the levels of *TIL* were classified as high when the H-score exceeded 160 and low if the H-score was less than 160.

The association between *FasL* and *TIL* classification was then analyzed using Chi-Square test (Table 2). Interestingly, there was a nine-fold odds ratio (OR) of lower *TILs* classification in high expression of* FasL* classification (OR 9, p=0.01) as presented in Table 2. The section, which showed high expression of* FasL*, tended to have lower level of *TILs*, and, reciprocally, sections with high level of *TILs* exhibited lower level of *FasL* expression. In other words, there was seemingly a negative correlations between the expression of* FasL* and the level of *TILs* in cervical cancer.

**Table 1:** Demographic Characteristics of the Subjects

Variable	n (%)
Age (years)	
<35 years	4 (12.5)
35-50 years	19 (59.4)
>50 years	9 (28.1)
Marital age (years)	
<16 years	11 (34.4)
16-24 years	18 (56.3)
>24 years	3 (9.4)
Marital frequency	
Once	29 (90.6)
Twice	1 (3.1)
Thrice	2 (6.3)
Parity	
1-2	19 (59.4)
3-5	9 (28.1)
≥6	4 (12.5)
Age at first labor	
<25 years	28 (87.5)
≥25 years	4 (12.5)
Contraceptive use	
Never	14 (43.8)
Pills	7 (21.9)
Injection	8 (25.0)
IUD	3 (9.4)
Pap smear history	
Never	31 (96.9)
Yes	1 (3.1)

**Table 2:** The Classification and Correlation of *FasL* Expression and *TIL* Level

	TILs Classification					*P*-value	OR
	High Level (>160)		Low Level (≤160)				
*FasL * Classification	n	(%)	n	(%)	Total		
High expression (≥140)	3	25	9	75	12	0.01	9
Low expression (<140)	15	75	5	25	20		
Total	18		14		32		

## Discussion

Based on a case-control study of cervical cancer patients in the USA, the number of pregnancies (parity) is positively correlated with higher relative risk of cervical cancer (Brinton et al., 1989). In addition, number of sexual partners is also positively correlated with higher odd ratios of cervical cancer in Thailand population (Chichareon et al., 1998). On the other hand, age at first intercourse is negatively correlated with the odds ratio, meaning that the older ones were at first intercourse, the smaller the odds ratio is for cervical cancer (Herrero et al., 2009; Plummer et al., 2014). Those findings were our basis in determining the demographic variables in this study, although they could not be coherently illustrated in our study, because we did not compare the data with controls. What can be consistently observed is of pap smear history, which showed that most of our patients (93,9%) had never had pap smear examination before, and pap smear examination has already been proven to be able to reduce the risk of cervical cancer (Chichareon et al., 1998; Vecchia et al., 1984).

In this study, we aimed to elucidate the association of FasL expression and the number of *TILs* in cervical cancer tissue since their correlation is quite controversial in a number of other cancers. Normally, immune cells employ FasL for defense mechanism to mediate the apoptosis of cancer cells and virus-infected cells. The binding of FasL on immune cells, such as cytotoxic-T and NK-cells, to the FasR of targeted cells stimulates the formation of death-inducing signaling complex which involves the adaptor molecule Fas-associated with a death domain (FADD), procaspase-8, procaspase-10, caspase-8/10 and regulator cellular FLICE inhibitory protein (c-FLIP). The recruitment of all these factors eventually leads to apoptosis of Fas-expressing cells (Walczak and Krammer, 2000).

Despite of *Fas/FasL* role in inducing apoptosis, most cancer cells are resistant to Fas-mediated apoptosis. There are several ways by which cancer cells avoid the *Fas/FasL-*induced apoptosis exerted by *TIL*. Firstly, cancer cells are capable of regulating the Fas trafficking to their cell surface by up-regulating *Fas-associated phosphatase-1* (*FAP-1*). High expression of *FAP-1* is associated with low expression of *Fas* on cell surface of melanoma cell line, whereas silencing *FAP-1* expression results in restored *Fas* expression on the cell membrane (Ivanov et al., 2003). Secondly, tumor cells can sabotage apoptotic signal in many different levels by increasing cFLIP (Irmler et al., 1997), reducing *FADD* expression (Tourneur et al., 2003), and attenuating caspase-8 expression (Teitz et al., 2000).

In this study, we found the inverse correlation between *FasL* expression on the cell surface of cancer cells and *TIL* counts. It has been reported that *FasL* upregulation in cancer cells can actually induce apoptosis in *TILs* as most *TILs* in cancer cells also express *Fas* and *TILs* themselves are prone to *Fas*-induced apoptosis. Another reason why we observed declined number of *TILs* is because not only cancer cells, but also endothelial cells in tumor tissue express *FasL*. It was demonstrated that *FasL*-expressing endothelial cells form a barrier for* TIL* resulting in the declined number of *TIL* that can actually infiltrate the tumor. Such upregulation of FasL in tumor endothelium is stimulated by the secretion of some factors by cancer cells including interleukin-10 (IL-10), prostaglandin E2, and vascular endothelial growth factor-2 (VEGF-2). It is noteworthy that the increased expression of FasL induces apoptosis only in effector T-cells, but not in regulatory T (T-reg) cells, indicating that* FasL* also contributes in attenuating the anti-tumor immunity. Syngeneic in vivo mouse model for ovarian cancer showed high expression of *FasL* on endothelial cells which led to decreased number of CD8+ T cells in tumor tissue. Accordingly, the inhibition of FasL using antibody resulted in increased infiltration of adoptively transferred tumor vaccine-primed CD8+ T cells (Motz et al., 2014; Zhu et al., 2018).

In addition to membrane FasL in tumor, *FasL* is also found in sera of patients suffering from various types of cancer such as ovarian, pancreatic and head and neck cancer (Walczak and Krammer, 2000). The presence of soluble *FasL *and, in some cases, also *Fas* in patients’ sera is associated with tumor growth, metastases and eventually with poor prognosis. *FasL* ability to promote tumor growth was confirmed by a study showing that *FasL* stimulation on apoptosis-resistant tumor cells activates downstream signaling cascade involving urokinase plasminogen activator, which ultimately induces motility and invasiveness. This finding is highly relevant with the fact that increased level of soluble *FasL* in patients’ sera was detected after the exposure of chemotherapy and might play an important role in treatment-resistance of the tumor (Barnhart et al., 2004).

This study demonstrated semi-quantitative* FasL *expressions in cervical cancer tissue, which could ilustrate the present state of the tissue compared to cell culture. Yet, this study did not take Fas expression and apoptosis into account that may describe the interaction within the whole extrinsic apoptosis system.

In conclusion, there is an inverse correlation between *FasL* expression and *TILs *levels that might indicate *FasL*-induced *TILs* apoptosis in tumor tissue. This association is shown between *FasL* expression (overexpression and non-overexpression) and *TILs* (levels and classification) in early stage cervical cancer (stage ≤ IIA2) that underwent radical hysterectomy.

The strong inverse correlation between *FasL* and *TILs* presence showed some insight about the interactions between cancer cells and its surroundings inside of the cervical cancer tissue. This might also be further developed to tailor a prognostic marker that can predict the outcome of therapy in patients, not only in cervical cancer, but generally in all cancer.
